# Supplementary material for: Modulatory action of Bryonia alba on the immune system in cyclophosphamide induced immunosuppression in BALB/c mice
Source: PLoS One. 2024 Dec 31;19(12):e0309756. doi: 10.1371/journal.pone.0309756 (PMC11687816; doi:10.1371/journal.pone.0309756)

PLOS ONE

Modulatory Action of Bryonia alba on the Immune System in Cyclophosphamide Induced Immunosuppression in BALB/c Mice

--Manuscript Draft--

**Manuscript Number:**	PONE-D-24-16624
**Article Type:**	Research Article
**Full Title:**	Modulatory Action of Bryonia alba on the Immune System in Cyclophosphamide Induced Immunosuppression in BALB/c Mice
**Short Title:**	Immunomodulation of Potentised Dilutions of Bryonia alba
**Corresponding Author:**	GV Narasimha Kumar<br>Dr Anjali Chatterji Regional Research Institute for Homoeopathy<br>Kolkata, INDIA
**Keywords:**	Immunocompromised Host; Bryonia alba; Chemotherapy; Blood Cell, Innate Immunity
**Abstract:**	Oxidative stress and inflammation are the most common pathologies in immune-compromised diseases and cancer treatments. The study examined the immune stimulation properties of Bryo-nia alba (BA) in different potencies (6C, 30C, and 200C) on a BALB/c mice model with a compro-mised immune system induced by cyclophosphamide (CPM) at a dose of 80 mg/kg. Seventy mice (35 males and 35 females) were randomly distributed into seven groups of 5 animals/sex. Mice treated with different potencies of BA showed notable improvements in various immune parameters, including RBC, WBC, and Hb levels, as well as thymus and phagocytic indices. Treatment also increased serum levels and splenic mRNA expression of IL-2, IL-4, TNF-α, and IFN-γ. The histopathology analysis showed that the spleen sections of the normal Group exhib-ited intact white and red pulp. In contrast, the sections of the CPM group exhibited disrupted and atrophied white pulp. The treatment with BA maintained the spleen in a preserved state, with the white and red pulp remaining intact and a higher density of lymphocytes. The results indi-cate that BA could serve as a valued immunostimulant agent when administered with chemo-therapy. However, additional research is required to assess the immunostimulatory effects of BA in immune-compromised infections.
**Order of Authors:**	Vara Prasad Saka
	GV Narasimha Kumar
	Abanti Goswami
	Bharat Kumar Reddy Sanapalli
	Pankaj Gupta
	Digvijay Verma
	Subhash Kaushik
**Additional Information:**	
**Question**	**Response**
**Financial Disclosure**<br><br>Enter a financial disclosure statement that describes the sources of funding for the work included in this submission. Review the submission guidelines for detailed requirements. View published research articles from *PLOS ONE* for specific examples.	Yes

Powered by Editorial Manager® and ProduXion Manager® from Aries Systems Corporation

This statement is required for submission and **will appear in the published article** if the submission is accepted. Please make sure it is accurate.<br><br>**Funded studies**<br>Enter a statement with the following details:<br>• Initials of the authors who received each award<br>• Grant numbers awarded to each author<br>• The full name of each funder<br>• URL of each funder website<br>• Did the sponsors or funders play any role in the study design, data collection and analysis, decision to publish, or preparation of the manuscript?<br><br>Did you receive funding for this work?	
Please add funding details.<br>as follow-up to "**Financial Disclosure**<br><br>Enter a financial disclosure statement that describes the sources of funding for the work included in this submission. Review the submission guidelines for detailed requirements. View published research articles from *PLOS ONE* for specific examples.<br><br>This statement is required for submission and **will appear in the published article** if the submission is accepted. Please make sure it is accurate.<br><br>**Funded studies**<br>Enter a statement with the following details:<br>• Initials of the authors who received each award<br>• Grant numbers awarded to each author<br>• The full name of each funder<br>• URL of each funder website<br>• Did the sponsors or funders play any role in the study design, data collection and analysis, decision to publish, or preparation of the manuscript?	Central Council for Research in Homoeopathy, India sponsored the study under the Intramural Research Scheme (4-13/2019-20/CCRH/Tech/DS/Kolkata/DSU/3255 dated 18-03-2021).

Powered by Editorial Manager® and ProduXion Manager® from Aries Systems Corporation

Did you receive funding for this work?"	
Please select the country of your main research funder (please select carefully as in some cases this is used in fee calculation).<br>as follow-up to "**Financial Disclosure**<br><br>Enter a financial disclosure statement that describes the sources of funding for the work included in this submission. Review the submission guidelines for detailed requirements. View published research articles from *PLOS ONE* for specific examples.<br><br>This statement is required for submission and **will appear in the published article** if the submission is accepted. Please make sure it is accurate.<br><br>**Funded studies**<br>Enter a statement with the following details:<br>• Initials of the authors who received each award<br>• Grant numbers awarded to each author<br>• The full name of each funder<br>• URL of each funder website<br>• Did the sponsors or funders play any role in the study design, data collection and analysis, decision to publish, or preparation of the manuscript?<br><br>Did you receive funding for this work?"	INDIA - IN
**Competing Interests**<br><br>Use the instructions below to enter a competing interest statement for this submission. On behalf of all authors, disclose any competing interests that could be perceived to bias this work—acknowledging all financial support and any other relevant financial or non-financial competing interests.<br><br>This statement is required for submission	The authors have declared that no competing interests exist.

and **will appear in the published article** if the submission is accepted. Please make sure it is accurate and that any funding sources listed in your Funding Information later in the submission form are also declared in your Financial Disclosure statement.<br><br>View published research articles from *PLOS ONE* for specific examples.<br><br>**NO authors have competing interests**<br><br>Enter: *The authors have declared that no competing interests exist.*<br><br>**Authors with competing interests**<br><br>Enter competing interest details beginning with this statement:<br><br>*I have read the journal's policy and the authors of this manuscript have the following competing interests: [insert competing interests here]*<br><br>* typeset	
**Ethics Statement**<br><br>Enter an ethics statement for this submission. This statement is required if the study involved:<br><br>• Human participants<br>• Human specimens or tissue<br>• Vertebrate animals or cephalopods<br>• Vertebrate embryos or tissues<br>• Field research<br><br>Write "N/A" if the submission does not require an ethics statement.<br><br>General guidance is provided below. Consult the submission guidelines for detailed instructions. **Make sure that all information entered here is included in the Methods section of the manuscript.**	All the animal studies were approved by the Institutional Animal Ethics Committee with proposal no. DACRRIH/CPCSEA/IAEC/ 2021/005. During the study, animals were housed, handled, and cared for in compliance with the CCSEA guidelines for Laboratory Animal Facility issued by the CCSEA, Govt of India.

**Format for specific study types**<br><br>**Human Subject Research (involving human participants and/or tissue)**<br>• Give the name of the institutional review board or ethics committee that approved the study<br>• Include the approval number and/or a statement indicating approval of this research<br>• Indicate the form of consent obtained (written/oral) or the reason that consent was not obtained (e.g. the data were analyzed anonymously)<br><br>**Animal Research (involving vertebrate animals, embryos or tissues)**<br>• Provide the name of the Institutional Animal Care and Use Committee (IACUC) or other relevant ethics board that reviewed the study protocol, and indicate whether they approved this research or granted a formal waiver of ethical approval<br>• Include an approval number if one was obtained<br>• If the study involved *non-human primates*, add *additional details* about animal welfare and steps taken to ameliorate suffering<br>• If anesthesia, euthanasia, or any kind of animal sacrifice is part of the study, include briefly which substances and/or methods were applied<br><br>**Field Research**<br><br>Include the following details if this study involves the collection of plant, animal, or other materials from a natural setting:<br>• Field permit number<br>• Name of the institution or relevant body that granted permission	
**Data Availability**<br><br>Authors are required to make all data underlying the findings described fully available, without restriction, and from the time of publication. PLOS allows rare exceptions to address legal and ethical concerns. See the PLOS Data Policy and FAQ for detailed information.	Yes - all data are fully available without restriction

A Data Availability Statement describing where the data can be found is required at submission. Your answers to this question constitute the Data Availability Statement and **will be published in the article**, if accepted.<br><br>**Important:** Stating 'data available on request from the author' is not sufficient. If your data are only available upon request, select 'No' for the first question and explain your exceptional situation in the text box.<br><br>Do the authors confirm that all data underlying the findings described in their manuscript are fully available without restriction?	
**Describe where the data may be found in full sentences. If you are copying our sample text, replace any instances of XXX with the appropriate details.**<br><br>• If the data are **held or will be held in a public repository**, include URLs, accession numbers or DOIs. If this information will only be available after acceptance, indicate this by ticking the box below. For example: *All XXX files are available from the XXX database (accession number(s) XXX, XXX.).*<br>• If the data are all contained **within the manuscript and/or Supporting Information files**, enter the following: *All relevant data are within the manuscript and its Supporting Information files.*<br>• If neither of these applies but you are able to provide **details of access elsewhere**, with or without limitations, please do so. For example:<br><br>*Data cannot be shared publicly because of [XXX]. Data are available from the XXX Institutional Data Access / Ethics Committee (contact via XXX) for researchers who meet the criteria for access to confidential data.*<br><br>*The data underlying the results presented in the study are available from (include the name of the third party*	All relevant data are within the manuscript and its Supporting Information files.

and contact information or URL).<br>• This text is appropriate if the data are owned by a third party and authors do not have permission to share the data.<br><br>* typeset	
Additional data availability information:	

Manuscript

Click here to access/download;Manuscript;Manuscript PONE.docx

Modulatory Action of *Bryonia alba* on the Immune System in Cyclophosphamide Induced Immunosuppression in BALB/c Mice

Short title: Immunomodulation of Potentised Dilutions of *Bryonia alba*

Vara Prasad Saka[1], Narasimha Kumar GV*[1], Abanti Goswami[1], Bharat Kumar Reddy Sanapalli[2], Pankaj Gupta[3], Digvijay Verma[4], Subhash Kaushik[4]

[1]Department of Pharmacology, Drug Standardization, Dr Anjali Chatterji Regional Research Institute for Homeopathy, Kolkata, West Bengal, INDIA.

[2] Department of Pharmacology, School of Pharmacy and Technology Management, SVKM's Narsee Monjee Institute of Management Studies (NMIMS) Deemed-to-be University, Jadcherla - 509301, Hyderabad, INDIA

Department of Pharmacology, Drug Standardization, Dr. D P Rastogi Central Research Institute for Homeopathy, Noida, Uttar Pradesh, INDIA.

[3]Drug Standardization, Central Council for Research in Homeopathy, New Delhi, INDIA.

***Corresponding author:**

Dr. G. V. Narasimha Kumar,

Department of Pharmacology, Drug Standardization,

Dr. Anjali Chatterji Regional Research Institute for Homeopathy,

Under Central Council for Research in Homeopathy,

Ministry of AYUSH, Kolkata, West Bengal, INDIA.

Phone no: +91 9949102404

Email: narasimha.spsp@gmail.com

Abstract

Oxidative stress and inflammation are the most common pathologies in immune-compromised diseases and cancer treatments. The study examined the immune stimulation properties of Bryo-nia alba (BA) in different potencies (6C, 30C, and 200C) on a BALB/c mice model with a compro-mised immune system induced by cyclophosphamide (CPM) at a dose of 80 mg/kg. Seventy mice (35 males and 35 females) were randomly distributed into seven groups of 5 animals/sex. Mice treated with different potencies of BA showed notable improvements in various immune parameters, including RBC, WBC, and Hb levels, as well as thymus and phagocytic indices. Treatment also increased serum levels and splenic mRNA expression of IL-2, IL-4, TNF-α, and IFN-γ. The histopathology analysis showed that the spleen sections of the normal Group exhib-ited intact white and red pulp. In contrast, the sections of the CPM group exhibited disrupted and atrophied white pulp. The treatment with BA maintained the spleen in a preserved state, with the white and red pulp remaining intact and a higher density of lymphocytes. The results indi-cate that BA could serve as a valued immunostimulant agent when administered with chemo-therapy. However, additional research is required to assess the immunostimulatory effects of BA in immune-compromised infections.

Key words: Immunocompromised Host; *Bryonia alba*; Chemotherapy; Blood Cell, Innate Immunity

Introduction

The human immune system is a robust defensive system that provides protection against pathogens and foreign antigens [1,2]. The immune response encompasses both innate and adaptive immunity, which relies on vital immunological organs like the thymus and spleen, along with immune cells that are essential for maintaining the body's immunity. Nevertheless, the immune system's defense activity can be affected by various factors such as age, metabolic disorders, stress-related conditions, and immune-related infections [3-5]. In recent times, it has been noted that a weakened immune system is a common symptom of a range of illnesses, including COVID-19 caused by SARS-CoV-2 [6]. In addition to that, the rise of infectious diseases such as HIV, Ebola hemorrhagic fever, SARC, Zika virus-related encephalopathy, COVID-19, and others, has sparked interest in studying immune-related infections [7].

Augmentation of the immune response is desirable to prevent infection in states of immune suppression, fight established infections, and with other diseased states. Since ancient times, millions worldwide have been using traditional medicinal systems to develop/boost their immunity [8]. Homeopathy is one such ancient popular AYUSH medicinal system that has been used by millions of people around the world. When administered based on symptom similarity, homeopathic medicines alter the state of immune homeostasis and stimulate the body's defense mechanisms to combat the pathogen or correct any immunodeficiency or hypersensitivity [9]. *Bryonia alba* (BA), a Homoeopathic remedy, has been clinically used to treat frontal pain, cough, peritonitis, serous tissue inflammation, typhoid, pneumonia, jaundice, rheumatism, and heart health. Additionally, brain conditions characterized by serous exudation and facial neuralgia have been treated with BA [10]. BA belongs to the Cucurbitaceae family, and its roots are employed in the preparation of mother tinctures.

BA in different potencies has been said to treat several ailments in the homeopathic medical system. Homeopathic Materia Medica lists BA roots for its antirheumatic, antiphlogistic, expectorant, and anti-anxiety activities [11]. It was noted that BA and several other homeopathic remedies helped treat influenza in 1920 during the Spanish flu [12,13]. BA 30C was discovered to be helpful as a genus epidemicus for chikungunya prevention, with a risk reduction of at least 20% in developing the illness [14]. These results back up the use of BA as an individual homeopathic treatment for chikungunya fever (C.F.) and chronic post-chikungunya arthritis (PCCA). Moreover, the most recent manuscript describes the efficacy of BA homeopathic therapy against COVID-19 caused by SARS-CoV-26. These reports of BA's success in treating viral infections point to its immunomodulatory and antioxidant characteristics, suggesting that BA may also enhance the immune status during immunosuppressed conditions. However, BA's immunostimulant properties in immunosuppressed situations have not yet been explored. Therefore, the current study aimed to examine the immunostimulant effects of BA in immunocompromised BALB/c mice.

Materials and methods

Reagents & Chemicals

Bryonia alba, and Dispensing Alcohol (90%) were procured from Hahnemann Publishing Co. Private Ltd, India.

Selection, randomization, and blinding of animals

All the animal studies were approved by the Institutional Animal Ethics Committee with proposal no. DACRRIH/CPCSEA/IAEC/ 2021/005. During the study, animals were housed, handled, and cared for in compliance with the CCSEA guidelines for Laboratory Animal Facility issued by the CCSEA, Govt of India.

Seventy mice (35 males and 35 females) were randomly assigned to seven groups (Group I - VII). Every Group comprised five animals of each sex for the study. The animals chosen for

the study had a body weight variation on the day of randomization that did not exceed 20%, which was around 18-20 g for each Group and sex. The randomization process was carried out using an excel sheet [15].

All the test compounds and vehicles were partially blinded to the researchers by coding the test compound. The medicines and their potencies were decoded during the phase of report drafting [15].

Dose selection

Singh *et al*. (2019) used 20 µL/100 g p.o. as a standard volume for oral administration of homeopathic medicines in albino rats for pharmacological evaluation [16]. Future, the same dose was converted to albino mice using virtual web-based software developed by Jahnavi et al. (2019) and was found to be 40 µL/100 g p.o., administered with distilled water in a 1:9 ratio [17]. These evidences made us use 40 µL/100 g p.o., in the current study.

Preparation of the BA potencies and vehicle

The potencies of BA were prepared using a standardized procedure in accordance with the Homoeopathic Pharmacopoeia of India (HPI). The BA dried powder was carefully macerated in a solution of 80.47% v/v alcohol, to obtain high-quality mother tincture. One ml of mother tincture was diluted in ninety-nine ml of ethyl alcohol (HPI grade) and succeeded ten times in getting Potency 1. The subsequent potencies were created by diluting one ml of potency 1 with ninety-nine ml of ethyl alcohol and giving ten jerks. Further, this procedure was repeated to obtain the potentized dilutions of 6C, 30C, and 200C [15,16].

Preparation of drug and vehicle stock solution

Stock solutions of BA and vehicle (Dispensing alcohol) were prepared by diluting 1 ml of potentized dilution (6C, 30C, and 200C) with 9 ml of double distilled water (1:9 ratio) for administration to experimental animals [17,18] and all the solutions were blinded until the

statistical analysis. Animals in all the groups were administered the respective interventions at a dose volume of 40 µL/100 g, p.o.

Investigational procedure

The animals were segregated into seven groups, involving 5 males and 5 females each. Then collectively, Group I received distilled water (D/W) (40 µL/100 g, p.o.) and served as normal control; Group II received D/W (40 µL/100 g, p.o.) and maintained as disease control or CPM control. Group III received dispensing alcohol (DA) (40 µL/100 g, p.o.) and served as vehicle control. Group IV served as standard control and received Levamisole (40 mg/kg, p.o.). Groups V, VI, and VII received the BA (40 µL/100 g, p.o.) in their potencies, i.e., 6C, 30C & 200C respectively. Except for Group I, all other groups received 80 mg/kg Cyclophosphamide (CPM) as a potent cytotoxic drug on days 4, 8 and 12 [18] intraperitoneally for immune suppression before their respective treatments.

Parameters of observation

Determination of RBC, WBC, and hemoglobin (Hb) count

The blood samples were collected 24 hr after the last dose administration by puncturing the retro-orbital plexus of the animals under anesthesia into the vials containing an appropriate amount of anticoagulant to prevent coagulation. A veterinary hematology analyzer (Genrui VH50) was employed to test the blood samples for enumerating RBC, WBC, and Hb.

Determination of spleen and thymus indexes

On the 14th day, 24 hr following the completion of the last dose, cervical dislocation and dissection were performed to obtain spleen and thymus from each Group. The obtained spleen and thymus were weighed, and the organ index was calculated using the following formula [19].

$$Spleen\ index\ \left(\frac{mg}{10g}\right) = Spleen\ weight\ (mg)/Body\ weight\ (g)\ X\ 100$$

$$Thymus\ index\ \left(\frac{mg}{10g}\right) = Thymus\ weight\ (mg)/Body\ weight\ (g)\ X\ 100$$

Phagocytic index assay

The carbon clearance test was performed to evaluate the effect of test medicines on the phagocytic activity of the reticuloendothelial system (RES). The test was carried out as described by Cheng et al. 2005 and Raj & Gothandam, 2015 [20,21], with minor modifications. On the 14th day of the study, mice were weighed and then injected with pre-warmed Indian liquid ink (0.1 mL/10 g B.W.) through the tail vein, following an interval of 2 hr after the last dose administration. Later, the blood was collected at 2- and 10-min interval from retro-orbital sinus. A 25 μL of blood was mixed with 2 mL of 0.1% sodium carbonate solution. The absorbance was recorded at 660 nm keeping the sodium carbonate solution as blank. Later, mice were euthanized, to excise the spleen, and liver and weighed immediately. The carbon clearance rate (κ) and the phagocytic index (α) were determined using the following formulas:

$$Rate\ of\ carbon\ clearance\ (\kappa) = (\log \mathrm{OD1} - \log \mathrm{OD2})\ /\ \mathrm{T2} - \mathrm{T1}$$

$$Rate\ of\ phagocytic\ index\ (\alpha) = 3\sqrt{\kappa}\ X\ Body\ Weight\ /\ (Liver\ weight + Spleen\ weight)$$

Determination of IL-2, TNF-α, IFN-γ, and IL-4 in serum, and spleen

Mouse-specific ELISA kits (Elabscience, USA) were used in carrying out tests for the determination of cytokine levels in the serum and spleen of the test animals. During the same, the manufacturer's protocol was strictly followed. The serum was obtained from the collected blood. For the other part of the experiment, the protocol explained by Han *et al.* 2019, with minor modifications was followed. After perfusion, part of the spleen was weighed accurately and homogenized in an appropriate extraction buffer (Cell lysis Buffer), maintaining the temperature at 4˚C throughout the process. The homogenate was cold centrifugated for 15-20

min at 3500 rpm. The supernatant was piped out, and further analysis was done using an ELISA reader (Bio Rad, USA) following manufacturer protocol [19].

Determination of the mRNA expression levels IL-2, TNF-α, IFN-γ, and IL-4 in the spleen

After 24 hr of last dose administration, on the 15th day, mice from each group were sacrificed for spleen samples. Later, the spleen samples were weighed and transferred into a pre-chilled microcentrifuge tube and placed on an ice box to avoid RNA degradation. Spleen samples were stored at -20ºC for further processing. A spleen tissue sample of ~100 mg was used for RNA extraction. RNA extraction was performed in accordance with the manufacturer's guidelines using the Aurum total RNA mini kit (Bio-Rad, USA). The quantification and verification of RNA purity were conducted using the NanoDrop™ One Microvolume UV-Vis Spectrophotometer. cDNA was synthesized from mRNA in accordance with the manufacturer's guidelines using the iScript cDNA synthesis kit (Bio-Rad, USA). cDNA was further diluted 10 times and stored for RT-PCR. The mRNA expression levels of cytokines [IL-2. IL-4, TNF-α, IFN-γ, and glyceraldehydes-3-phosphate dehydrogenase (GAPDH)] were determined by real-time quantitative polymerase chain reaction (qPCR). GAPDH, a housekeeping gene was used as an internal control for cytokines expression to account for variations in mRNA loading. The cytokine's (IL-2. IL-4, TNF-α, IFN-γ, and GAPDH) primers used were detailed in Table 1. The iTaq Universal SYBR Green Supermix kit (Bio-Rad, USA) was used for the real-time PCR reaction. Real-time PCR was performed in a Bio-Rad CFX96 Real-Time fluorescent quantitative PCR detection system (Bio-Rad, USA). The thermal cycle condition used for reverse transcription was as follows: pre-denaturation program (3 min at 95°C), amplification and quantification program (5 s at 95°C, 20 s at 59.6°C) with 40 cycles, and melting curve program (0.05 s at 65°C, 5 s at 95°C). The relative quantification of objective genes was calculated according to the $2^{-\Delta\Delta Ct}$ method [22].

Table 1: Primer sequence for rtPCR

Gene	Sequence 5' - 3'	Size bp
IL-2	F-GCAGCTGTTGATGGACCTAC	20
	R-TCCACCACAGTTGCTGACTC	20
IL-4	F-TCGGCATTTTGAACGAGGTC	20
	R-GAAAAGCCCGAAAGAGTCTC	20
TNF-α	F-ATGAGCACAGAAAGCATGATC	21
	R-TACAGGCTTGTCACTGGAATT	21
IFN-γ	F-TGAGCAGAGCTCTTGTGGTC	20
	R-CGTTCCTCCTTGTGGCCTAA	20
GAPDH	F-5GTGGAGTCTACTGGTGTCTTC3	21
	R-5'GTGCAGGAGGCATTGCTTACA3'	21

Histopathological analysis of the spleen

After collection of the whole spleen, it was washed in sterile PBS (pH 7.3 ± 0.1), and without much delay, it was kept in buffered formalin solution for 24 hr. After the specified duration, the spleen was trimmed into 3-5 mm thick pieces for final sectioning. After proper fixation, the tissue was processed in increasing concentrations of alcohol, and completely dehydrated tissue was processed in xylene before it was embedded in liquid paraffin to prepare paraffin-tissue blocks. Using a semi-auto rotary microtome (CIM 18, Biocraft), specimen slides with 3-5 µm thick tissue sections were prepared. These slides were stained with hematoxylin and eosin (H&E) to analyze the pathophysiology of the spleen [23]. The images were captured under the magnification of 200X and 400X using a phase contrast microscope (Bx53, Olympus).

Statistical analysis

The data are presented as the means ± SEM. One-way ANOVA and Tukey's multiple comparison tests were conducted to compare multiple groups. All statistical analysis was

conducted using the SPSS 21 software. Statistical significance was determined based on values of $p<0.05$, 0.01, and 0.001.

Results

Effect of BA on haematological parameters

Initially, the RBC, WBC and Hb counts were assessed in the CPM induced immune compromised animals and found to be significantly lower ($P<0.001$) compared to the normal control animals indicating the induction of immunosuppression by CPM. Later, the samples from the BA treated animals in their potencies of 6C, 30C and 200C, showed significantly increased Hb and RBC levels ($p<0.001$) in comparison with those of CPM and vehicle treated groups. In case of WBC, BA 6C has shown a significant improvement i.e., $P<0.01$ and $P<0.05$ in comparison with those in CPM and vehicle treated groups respectively. Whereas, BA 200C showed significance of $P<0.05$ compared with the CPM and no significance with vehicle treated Group. The above findings indicates that the activity observed is due to the presence of BA but not because of vehicle used, i.e., dispensing alcohol. Levamisole treatment also significantly improved, Hb, RBC ($P<0.001$) and WBC ($P<0.01$) levels compared to the CPM control. The results indicate that BA could reverse CPM induced suppression of Hb, RBC, and WBC in mice (Fig. 1).

Fig 1: Effect of BA on Haematological parameters in CPM-Treated Mice; A-Heamoglobin, B- RBC count, C-WBC count; Data values are expressed as Mean ± SEM. n=10 in each group. Statistical analysis was performed using one-way ANOVA followed by Tukey multiple comparison post hoc test. $^{aaa}P<0.001$ when compared to Normal Control; $^{b}P<0.05$, $^{bb}P<0.01$, $^{bbb}P<0.001$ as compared to the CPM group; $^{c}P<0.05$, $^{cc}P<0.01$, $^{ccc}P<0.001$ as compared to the vehicle control group.

Effect of BA on spleen and thymus indices

In mice treated with CPM, spleen and thymus indices significantly decreased ($P<0.05$ and $P<0.001$, respectively) compared to the normal control. BA 30C ($P<0.001$) potency significantly improved the spleen index in immunocompromised BALB/c mice. BA 200C potency alone significantly improved ($P<0.05$) the thymus index, and 6C, 30C improvement were statistically non-significant. BA 30C significantly differed ($P<0.05$) from the vehicle-treated Group in improving spleen index, in comparison with vehicle treated Group. Levamisole-treated mice showed a significant improvement in the spleen ($P<0.001$) and thymus ($P<0.001$) indices than CPM control group. The above data suggested that BA could partially restore CPM induced reduction in spleen and thymus indices (Fig. 2A and 2B).

Effect of BA on the phagocytic index

Similar to the immune organ indices, the phagocytic index of the CPM treated animals was reduced significantly ($P<0.001$) compared to the normal control group. BA 6C ($P<0.01$), 30C ($P<0.001$), 200C ($P<0.05$) and levamisole ($P<0.05$) had improved phagocytic function in comparison to CPM control group (Fig. 2C).

Fig 2: Effect of BA on Immune Indices in CPM-Treated Mice; A- Spleen index, B-Thymus Index, C-Phagocytic index; Data values are expressed as Mean ± SEM. n=10 in each group. Statistical analysis was performed using one-way ANOVA followed by Tukey multiple comparison post hoc test. $^{aaa}P<0.001$ when compared to Normal Control; $^{b}P<0.05$, $^{bb}P<0.01$, $^{bbb}P<0.001$ as compared to the CPM group; $^{c}P<0.05$, $^{cc}P<0.01$, $^{ccc}P<0.001$ as compared to the vehicle control group.

Effect of BA on cytokine levels in serum and spleen

CPM treatment caused a significant reduction in the levels of IL-2, IL-4, TNF-α, and IFN-γ ($P<0.05$/$P<0.001$) in the serum and spleen tissue of mice than normal control, indicating its immunosuppressant effect. The BA 6C treatment has not showed any significant increase in the IL-2 levels of serum and spleen in comparison with CPM control. However, the BA 6C

has showed significance ($P<0.05$) in the serum IL-2 levels with no significance in spleen IL-2 levels in comparison with vehicle control. The BA 30C treatment has showed significant ($P<0.001$) increase of IL-2 levels in both serum and spleen in comparison with the CPM and vehicle control. BA 200C treatment has demonstrated a significant ($P<0.001$) rise in IL-2 levels in both serum and spleen, when compared to the vehicle control group. The BA 200C treatment has displayed no significance in serum IL-2 levels with significant ($P<0.001$) increase in spleen IL-2 levels in comparison with CPM control.

The BA 6C, 30C and 200C have displayed a significant ($P<0.001$, $P<0.001$ and $P<0.05$) increase in IL-4 levels in serum and spleen ($P<0.001$) compared to those in CPM and vehicle control groups.

The BA 6C and BA 200C did not demonstrate any significant rise in serum TNF-α levels. However, the BA 30C exhibited a significant ($P<0.001$) increase in serum TNF-α levels when compared to the CPM and vehicle control groups. In regards to the spleen TNF-α levels, it is worth noting that BA 6C did not demonstrate any significant findings. However, both BA 30C and 200C exhibited a significant increase ($P<0.001$) when compared to the CPM and vehicle control groups.

The BA 6C and 200C has showed significant ($P<0.001$) increase in the serum IFN-γ levels whereas BA 30C has not showed any significance in comparison with CPM and vehicle control. Coming to the spleen IFN-γ levels, BA 6C, 30 C and 200C has showed no significance in comparison with CPM and vehicle control.

For the positive control (Levamisole), it significantly enhanced ($P<0.001$) the secretions of serum IL-2, IL-4, IFN-γ and splenic levels of IL-4, TNF-α, and IFN-γ when compared to the CPM control group. These results indicate that BA could enhance immune activity by improving cytokines production in serum and splenocytes of immunosuppressed mice (Fig 3). **Fig 3: Effect of BA on Serum and Spleen cytokine levels in CPM-Treated Mice;** Data

values are expressed as Mean ± SEM. n=5 in each group. Statistical analysis was performed using one-way ANOVA followed by Tukey multiple comparison post hoc test. $^{aaa}P<0.001$ when compared to Normal Control; $^{b}P<0.05$, $^{bb}P<0.01$, $^{bbb}P<0.001$ as compared to the CPM group; $^{c}P<0.05$, $^{cc}P<0.01$, $^{ccc}P<0.001$ as compared to the vehicle control group.

Effect of BA on mRNA expression levels of the cytokines (IL-2, IL-4, TNF-α, and IFN-γ) in spleen

To further confirm the splenic immune enhancement by BA, qRT-PCR was performed to examine the induction of transcriptional upregulation of cytokines in the spleen. As shown in fig. 4, BA significantly increased (P<0.001) the mRNA levels of IL-2, IL-4, TNF-α, and IFN-γ while CPM-treated mice produced substantially lower (P<0.001) mRNA of levels of the above cytokines. Levamisole treatment also significantly upregulated (P<0.001) the mRNA expression of IL-2, IL-4, TNF-α, and IFN-γ genes compared to CPM-treated mice. The mRNA expression of the above cytokines was significantly different between BA and vehicle-treated groups, further confirming that BA was responsible for reversing the downregulation of mRNA expressions of cytokines induced by CPM in mice.

Fig 4: Effect of BA on mRNA expression levels of the cytokines in the spleen of CPM-Treated Mice; Data values are expressed as Mean ± SEM. n=10 in each group. Statistical analysis was performed using one-way ANOVA followed by Tukey multiple comparison post hoc tests. $^{aaa}P<0.001$ when compared to Normal Control; $^{b}P<0.05$, $^{bb}P<0.01$, $^{bbb}P<0.001$ as compared to the CPM group; $^{c}P<0.05$, $^{cc}P<0.01$, $^{ccc}P<0.001$ as compared to the vehicle control group.

Effect of BA on histology of spleen

The histology of spleen sections from normal mice stained with HE revealed distinct regions of white pulp and red pulp, with the former containing lymphoid tissue and the latter containing splenic cords and venous sinuses, as shown in fig. 5. However, treatment with

CPM resulted in a depletion or disruption of the white pulp, with a reduction in germinal centre formation and lymphocyte populations. This was accompanied by an increase in cellularity in the red pulp due to infiltration by immune cells. Additionally, alterations in the structural integrity of the spleen, with fibrosis in the tissue, were observed. The histoarchitecture of the spleen in immunocompromised mice was not protected by alcohol treatment and instead led to white pulp degeneration and lymphocyte infiltration into the red pulp. On the other hand, treatment with BA in its 6C, 30C, and 200C potencies showed a significant improvement in the histology of the spleen. The arrangement of the red and white pulp improved, indicating the protection from CPM induced damage. Similar protective effect was observed in case of mice treated with levamisole.

Figure 4: Effect of BA on mRNA expression levels of the cytokines in the spleen of CPM-Treated Mice; Data values are expressed as Mean ± SEM. n=10 in each group. Statistical analysis was performed using one-way ANOVA followed by Tukey multiple comparison post hoc tests. $^{aaa}P<0.001$ when compared to Normal Control; $^{b}P<0.05$, $^{bb}P<0.01$, $^{bbb}P<0.001$ as compared to the CPM group; $^{c}P<0.05$, $^{cc}P<0.01$, $^{ccc}P<0.001$ as compared to the vehicle control group.

Discussion

There is limited scientific evidence to support the use of BA as an immunomodulatory agent. Some studies have suggested that BA may have anti-inflammatory properties, which could potentially help to regulate the immune system.

In this scientific study, the focus was on the potential of BA ultra-high dilutions 6C, 30C & 200C to stimulate the immune system in CPM induced immune suppressed BALB/c mice. CPM is an effective chemotherapeutic drug used in the treatment of various tumors, but it is also known to have a range of negative side effects, including immunosuppression, myelosuppression, and oxidative stress [24]. The immunosuppression resulting from CPM can

leave the body vulnerable to infection and other external pathogens. Furthermore, CPM challenge can cause damage and weight loss in the spleen and thymus, which, as representative immune organs, play an essential role in maintaining the body's immune homeostasis [25].

To induce immunosuppression, mice from all groups aside from the normal control group were intraperitoneally given CPM at a dose of 80 mg/kg on days 4, 8, and 12 of the study. Our results indicate that CPM significantly reduced the RBC, WBC, and H.B. levels along with the spleen and thymus indices in comparison with normal control group. The concentrations of IL-2, IL-4, TNF-α, and IFN-γ in the serum and spleen were also reduced by CPM. In addition, CPM mice showed a marked decline in splenic mRNA expression of IL-2, IL-4, TNF-α, and IFN-γ. According to earlier studies and the results presented above, an immunosuppressive mice model using CPM was successful [17,26,27].

The normal ranges of various animal white blood cells varied. A higher or lower than normal count could be indicative of a number of illnesses, such as rheumatoid arthritis, allergies, bone marrow deficiencies, specific viral infections, or severe bacterial infections [28]. CPM administration resulted in a significant reduction of RBC, WBC and Hb levels in mice. BA (6C, 30C, and 200C) treatment significantly improved the decline of RBC and H.B. levels induced by CPM in mice. BA at 6C and 200C also significantly improved the WBC count in CPM treated mice. The results indicate that BA treatment could enhance the immunity in mice by improving RBC, WBC & Hb levels reduced by CPM.

The body's immune system includes the thymus and spleen, two crucial organs [15]. Reduced size and weight of the spleen and thymus in pathological conditions indicate a decline in immune response [29]. In this study, BA treatment partially improved spleen and thymus indices compared to the CPM control without any statistical significance, suggesting that BA may act to prevent the immune organ atrophy brought on by CPM.

The macrophage function was assessed by carbon clearance test. When serum protein-bound particles of Indian ink containing carbon injected directly into the systemic circulation, macrophages in the liver and spleen intensely swallowed the particles. The rate at which macrophages removed carbon from the blood was determined by an exponential equation, and phagocytic activity was positively correlated with the rate at which carbon particles were removed [17]. Our study implies that the macrophage system's phagocytic activity was reduced by CPM therapy, which concurs with earlier studies [30,31]. BA treatment had insignificant effects on the phagocytic activity of macrophages compared with the CPM control.

The impact of BA on the synthesis of IL-2, IL-4, TNF-α, and IFN-γ was assessed because cytokines are crucial for triggering an immune response. Numerous cellular and animal studies also demonstrated that enhancing cytokine synthesis enhance immune function in immunodeficient model mice [17,28,30,32,33]. Cytokines function as a signalling molecule among cells that control the immune response, mediate inflammatory responses, participate in immune cell differentiation, and promote hematopoietic function [34]. A cell growth factor called IL-2 encourages cell division and proliferation. Interferon production and inflammatory or autoimmune reactions can both be facilitated by IL-2. TNF-α is a crucial component of the host defence system and kills tumour cells. Additionally, TNF-α can promote the expression of a number of inflammatory and immune mediators. The biological activities of IFN-γ are primarily to activate macrophages, inhibit viral replication, and induce the expression of MHC molecules. Th2, mast cells, and N.K. cells secrete IL-4, which interacts with T, B, macrophages, and mast cells to activate and differentiate B cells, increase class II MHC expression, and induce and grow T cells and mast cells, respectively [18,31,35]. Our study results show that BA had an excellent regulatory effect on cytokines production in CPM induced immunosuppressed mice. BA at 30C raised the concentrations of IL-2, TNF-α,

and IL-4 in mouse serum, 30C and 200C potencies raised the levels of the IL-2, TNF-α, and IL-4 in the splenic cytokines indicating the effect of BA on splenocytes also. This finding supports BA's ability to balance the body's immune system and lessen immunosuppression symptoms.

qRT-PCR was performed on spleen cytokines, such as IL-2, IL-4, TNF-α, and IFN-γ, to further support the immune enhancement of BA in the spleen from a CPM-induced immunosuppressed state. The results revealed a significant increase in the mRNA levels of IL-2, IL-4, TNF-α, and IFN-γ in the spleen of BA treated mice, compared to the CPM group, which produced lower levels of these cytokines. Through the current investigation, it was revealed that BA had significantly improved the cytokine production in mice undergoing immunosuppression with CPM, displaying moderate yet noteworthy immunostimulant potency.

The histological stained sections of spleen exhibited interesting data that are in good agreement with the existing piece of evidences. The normal group displayed intact white and red pulp proving that the spleen is in good health. The CPM treated group displayed disruption, degeneration and atrophy in the spleen, indicating its potential harm [17,26,27]. However, the BA administration demonstrated a protective impact by exhibiting intact white and red pulp with well-defined borders and a higher concentration of lymphocytes in the spleen. Levamisole treatment also displayed similar positive effects.

To summarize, the study emphasizes the therapeutic benefits of BA in safeguarding the spleen against damage caused by chemotherapy. The findings also indicate that alcohol, when used as a vehicle for BA, does not impact its immunostimulant properties, as demonstrated by the lack of improvement in CPM-treated mice. Additionally, the study found that the immunoenhancement activity of BA potencies (6C, 30C, and 200C) is comparable to that of the standard immunostimulant control, Levamisole. These results provide a solid foundation

for future investigations into the immunomodulatory potential of BA clinically, which could have important implications for the development of homeopathic medicines as immunomodulatory agents.

The study's limitations encompass the utilization of a mice model to simulate chemotherapy-induced immunosuppression through CPM administration. While this model is widely employed, it might not entirely replicate the intricate immunological responses observed in human chemotherapy patients. Despite the assessment of cytokine levels and immune cell markers, a deeper exploration of BA intricate immunomodulatory effects, including the investigation of molecular signaling pathways and cellular interactions, could provide valuable insights into its mode of action. Despite these constraints, the research underscores BA potential therapeutic benefits in mitigating chemotherapy-induced immune system impairment. It establishes a foundation for future inquiries into BA immunomodulatory properties and its clinical significance in immune-related conditions. Moreover, the study's methodology and approach could serve as a blueprint for exploring BA immunomodulatory potential in diverse immune-related disorders beyond chemotherapy-induced immunosuppression.

Conclusion

The present study proves, for the first time, that extremely high dilutions of BA 6C, 30C, and 200C potencies used in the homeopathic system of medicine can lessen the adverse effects of CPM on immune function in immune compromised mice. Based on the results, though the precise underlying mechanism of BA is unknown, we conclude that BA's ability to increase RBC and WBC levels, spleen index, IL-2, IL-4, TNF-α, and IFN-γ cytokine levels in serum & spleen are responsible for the immune stimulant activity it imparts. Above all, our research may offer a mechanistic rationale for using BA -6C, 30C, and 200C as effective adjuvant immunostimulant therapy or a different strategy to lessen CPM induced immunosuppression.

The study results encourage further investigations to evaluate the immunostimulant potential of BA in immune compromised conditions brought on by infections.

Availability of data and material

The study material is stored in the Archive of the test facility, Dr. Anjali Chatterji Regional Research Institute for Homeopathy. The study material includes electronic backup and print copies of the Study Plan, Raw Data, Microsoft Excel compiled raw data, Statistical output data, and Final Report. The materials will be retained for at least 05 years from the archival of the final report. After that, as per the directions of the competent authority, the archived materials may be disposed of, or the archival period may be extended.

Funding

Central Council for Research in Homoeopathy, India sponsored the study under the Intramural Research Scheme (4-13/2019-20/CCRH/Tech/DS/Kolkata/DSU/3255 dated 18-03-2021).

Authors contributions

Vara Prasad Saka, and **Abanti Goswami**: Investigation and Formal analysis. **Narasimha Kumar GV** and **Pankaj Gupta**: Conceptualization, Supervision and Methodology. **Bharat Kumar Reddy. Sanapalli**: Writing-Reviewing & Editing and Visualization. **Digvijay Verma**: Project administration. **Subhash Kaushik**: Funding acquisition and Writing-Reviewing & Editing. All authors read and approved the manuscript.

References

1. Kajaria, D.; Tripathi, J.S.; Tiwari, S.K.; Pandey, B.L. Immunomodulatory effect of ethanolic extract of Shirishadi compound. *Ayu* **2013**, *34*, 322.
2. Chaplin, D.D. Overview of the immune response. *Journal of allergy and clinical immunology* **2010**, *125*, S3-S23.

3. Grubeck-Loebenstein, B. Changes in the aging immune system. *Biologicals* **1997**, *25*, 205-208.
4. Beissert, S.; Schwarz, T. Mechanisms involved in ultraviolet light-induced immunosuppression. In Proceedings of the Journal of Investigative Dermatology Symposium Proceedings, 1999; pp. 61-64.
5. Wustrow, T.; Mahnke, C. Causes of immunosuppression in squamous cell carcinoma of the head and neck. *Anticancer research* **1996**, *16*, 2433-2468.
6. Diao, B.; Wang, C.; Tan, Y.; Chen, X.; Liu, Y.; Ning, L.; Chen, L.; Li, M.; Liu, Y.; Wang, G. Reduction and functional exhaustion of T cells in patients with coronavirus disease 2019 (COVID-19). *Frontiers in immunology* **2020**, 827.
7. Hickman, H.D.; Suthar, M.S. Editorial overview: Viral immunology: Generating immunity to diverse viral pathogens. *Current opinion in virology* **2018**, *28*, viii.
8. Ahmad, W.; Jantan, I.; Kumolosasi, E.; Bukhari, S.N.A. Immunostimulatory effects of the standardized extract of Tinospora crispa on innate immune responses in Wistar Kyoto rats. *Drug design, development and therapy* **2015**, 2961-2973.
9. Poitevin, B.; Davenas, E.; Benveniste, J. In vitro immunological degranulation of human basophils is modulated by lung histamine and Apis mellifica. *British journal of clinical pharmacology* **1988**, *25*, 439-444.
10. Srivastava, T.N. Flora gorakhpurensis. *(No Title)* **1976**.
11. Boericke, W. Homöopathic Materia Medica by William Boericke. *Englische Erstauflage* **1927**.
12. Shinde, V. Homoeopathy in pandemic Spanish flu 1918. *Indian Journal of Research in Homoeopathy* **2020**, *14*, 152-159.
13. Baker, W. Research work in Gelsemium sempervirens and Bryonia alba in influenza. *J Am Inst Homeopath* **1920**, *12*, 695-698.

14. Janardanan Nair, K.; Gopinadhan, S.; Sreedhara Kurup, T.; Kumar, B.S.; Aggarwal, A.; Varanasi, R.; Nayak, D.; Padmanabhan, M.; Oberai, P. Homoeopathic Genus Epidemicus′ Bryonia alba′ as a prophylactic during an outbreak of Chikungunya in India: A cluster-randomised, double-blind, placebo-controlled trial. *Indian Journal of Research in Homoeopathy* **2014**, *8*, 160-165.

15. Bespalov, A.; Wicke, K.; Castagné, V. Blinding and randomization. *Good research practice in non-clinical pharmacology and biomedicine* **2019**, 81.

16. Singh, S.; Karwasra, R.; Kalra, P.; Nayak, D.; K Khurana, A.; K Manchanda, R. Attenuation of Complete Freund's Adjuvant-induced arthritis by different dilutions of Eupatorium perfoliatum and Crotalus horridus and their safety evaluation. **2019**.

17. Janhavi, P.; Divyashree, S.; Sanjailal, K.; Muthukumar, S. DoseCal: a virtual calculator for dosage conversion between human and different animal species. *Archives of Physiology and Biochemistry* **2022**, *128*, 426-430.

18. Winkelstein, A. Mechanisms of immunosuppression: effects of cyclophosphamide on cellular immunity. *Blood* **1973**, *41*, 273-284.

19. Han, L.; Meng, M.; Guo, M.; Cheng, D.; Shi, L.; Wang, X.; Wang, C. Immunomodulatory activity of a water-soluble polysaccharide obtained from highland barley on immunosuppressive mice models. *Food & function* **2019**, *10*, 304-314.

20. Cheng, W.; Li, J.; You, T.; Hu, C. Anti-inflammatory and immunomodulatory activities of the extracts from the inflorescence of Chrysanthemum indicum Linne. *Journal of ethnopharmacology* **2005**, *101*, 334-337.

21. Raj, S.; Gothandam, K. Immunomodulatory activity of methanolic extract of Amorphophallus commutatus var. wayanadensis under normal and cyclophosphamide induced immunosuppressive conditions in mice models. *Food and Chemical Toxicology* **2015**, *81*, 151-159.

22. Livak, K.J.; Schmittgen, T.D. Analysis of relative gene expression data using real-time quantitative PCR and the 2− ΔΔCT method. *methods* **2001**, *25*, 402-408.

23. Halder, S.; Sarkar, M.; Dey, S.; Bhunia, S.K.; Koley, A.R.; Giri, B. Protective effects of red grape (Vitis vinifera) juice through restoration of antioxidant defense, endocrine swing and Hsf1, Hsp72 levels in heat stress induced testicular dysregulation of Wister rat. *Journal of Thermal Biology* **2018**, *71*, 32-40.

24. Wang, H.; Wang, M.; Chen, J.; Tang, Y.; Dou, J.; Yu, J.; Xi, T.; Zhou, C. A polysaccharide from Strongylocentrotus nudus eggs protects against myelosuppression and immunosuppression in cyclophosphamide-treated mice. *International immunopharmacology* **2011**, *11*, 1946-1953.

25. Tanahashi, T.; Sekiguchi, N.; Matsuda, K.; Matsumoto, A.; Ito, T.; Nakazawa, H.; Ishida, F. A screening method with lymphocyte percentage and proportion of granular lymphocytes in the peripheral blood for large granular lymphocyte (LGL) leukemia. *International journal of hematology* **2017**, *105*, 87-91.

26. Zhou, X.; Dong, Q.; Kan, X.; Peng, L.; Xu, X.; Fang, Y.; Yang, J. Immunomodulatory activity of a novel polysaccharide from Lonicera japonica in immunosuppressed mice induced by cyclophosphamide. *PloS one* **2018**, *13*, e0204152.

27. Zhang, W.-N.; Gong, L.-L.; Liu, Y.; Zhou, Z.-B.; Wan, C.-X.; Xu, J.-J.; Wu, Q.-X.; Chen, L.; Lu, Y.-M.; Chen, Y. Immunoenhancement effect of crude polysaccharides of Helvella leucopus on cyclophosphamide-induced immunosuppressive mice. *Journal of Functional Foods* **2020**, *69*, 103942.

28. Gao, S.; Hong, H.; Zhang, C.; Wang, K.; Zhang, B.; Han, Q.-a.; Liu, H.; Luo, Y. Immunomodulatory effects of collagen hydrolysates from yak (Bos grunniens) bone

on cyclophosphamide-induced immunosuppression in BALB/c mice. *Journal of Functional Foods* **2019**, *60*, 103420.

29. Baqai, F.P.; Gridley, D.S.; Slater, J.M.; Luo-Owen, X.; Stodieck, L.S.; Ferguson, V.; Chapes, S.K.; Pecaut, M.J. Effects of spaceflight on innate immune function and antioxidant gene expression. *Journal of applied physiology* **2009**, *106*, 1935-1942.
30. Mei, Y.-x.; Chen, H.-x.; Zhang, J.; Zhang, X.-d.; Liang, Y.-x. Protective effect of chitooligosaccharides against cyclophosphamide-induced immunosuppression in mice. *International Journal of Biological Macromolecules* **2013**, *62*, 330-335.
31. Hou, H.; Fan, Y.; Wang, S.; Si, L.; Li, B. Immunomodulatory activity of Alaska pollock hydrolysates obtained by glutamic acid biosensor–Artificial neural network and the identification of its active central fragment. *Journal of functional foods* **2016**, *24*, 37-47.
32. Woo, S.M.; Choi, W.R.; Jang, D.; Yi, C.S.; Kim, H.L.; Kim, K.H.; Kim, J.T.; Choi, W.H.; Jang, S.H.; Kim, M.J. Immune enhancement effect of an herb complex extract through the activation of natural killer cells and the regulation of cytokine levels in a cyclophosphamide-induced immunosuppression rat model. *Asian Pacific Journal of Tropical Medicine* **2018**, *11*, 653-658.
33. Kumar, V.P.; Venkatesh, Y.P. Alleviation of cyclophosphamide-induced immunosuppression in Wistar rats by onion lectin (Allium cepa agglutinin). *Journal of ethnopharmacology* **2016**, *186*, 280-288.
34. Dirchwolf, M.; Podhorzer, A.; Marino, M.; Shulman, C.; Cartier, M.; Zunino, M.; Paz, S.; Muñoz, A.; Bocassi, A.; Gimenez, J. Immune dysfunction in cirrhosis: distinct cytokines phenotypes according to cirrhosis severity. *Cytokine* **2016**, *77*, 14-25.

35. Meng, Y.; Li, B.; Jin, D.; Zhan, M.; Lu, J.; Huo, G. Immunomodulatory activity of Lactobacillus plantarum KLDS1. 0318 in cyclophosphamide-treated mice. *Food & Nutrition Research* **2018**, *62*.

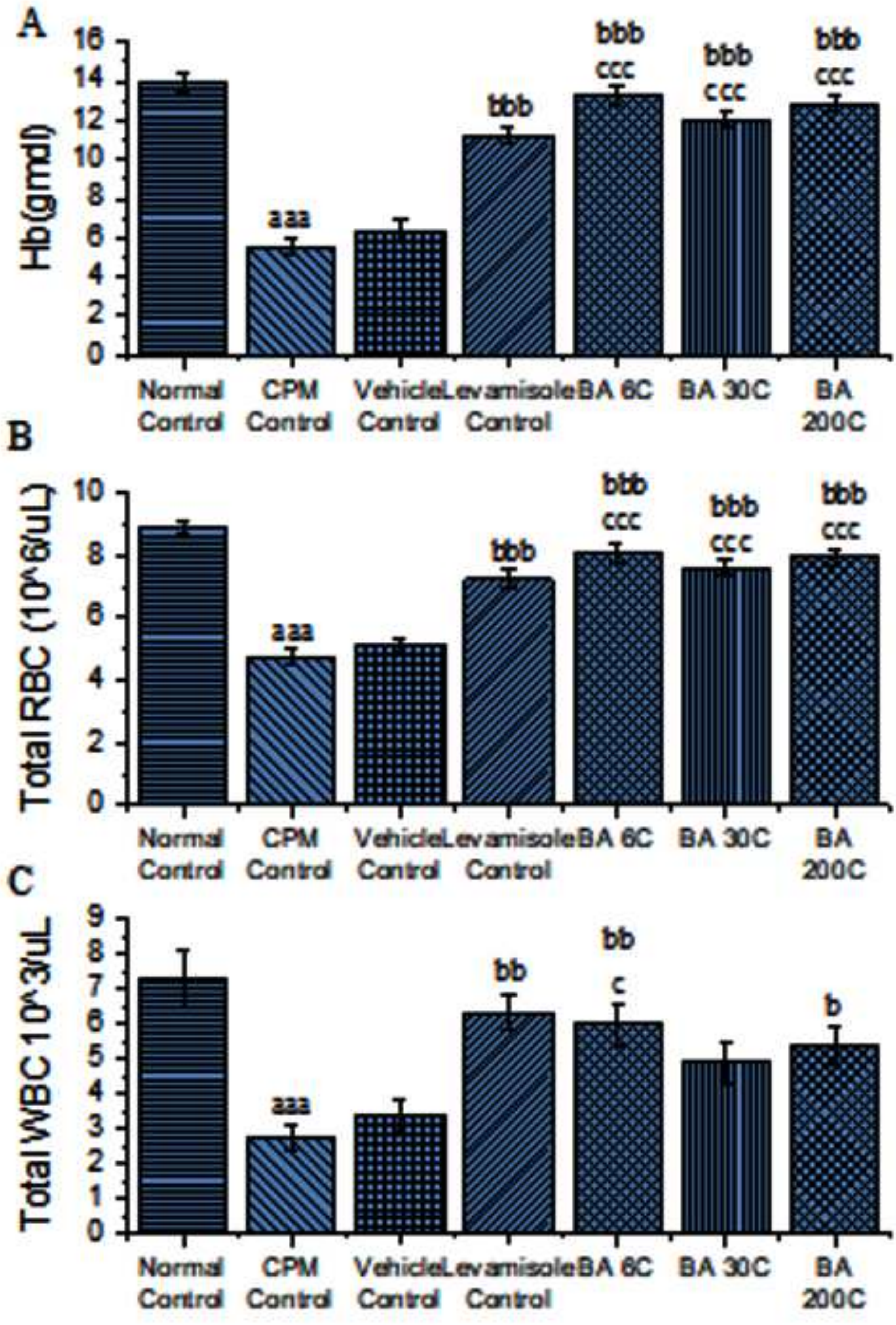
A
16
14
12
10
8
6
4
2
0
Hb(gmdl)
aaa
bbb
bbb
ccc
bbb
ccc
bbb
ccc
Normal Control
CPM Control
Vehicle Control
Levamisole Control
BA 6C
BA 30C
BA 200C
B
10
8
6
4
2
0
Total RBC (10^6/uL)
aaa
bbb
bbb
ccc
bbb
ccc
bbb
ccc
Normal Control
CPM Control
Vehicle Control
Levamisole Control
BA 6C
BA 30C
BA 200C
C
9
8
7
6
5
4
3
2
1
0
Total WBC 10^3/uL
aaa
bb
bb
c
b
Normal Control
CPM Control
Vehicle Control
Levamisole Control
BA 6C
BA 30C
BA 200C

Fig 2

Click here to access/download;Figure;Fig 2.tif

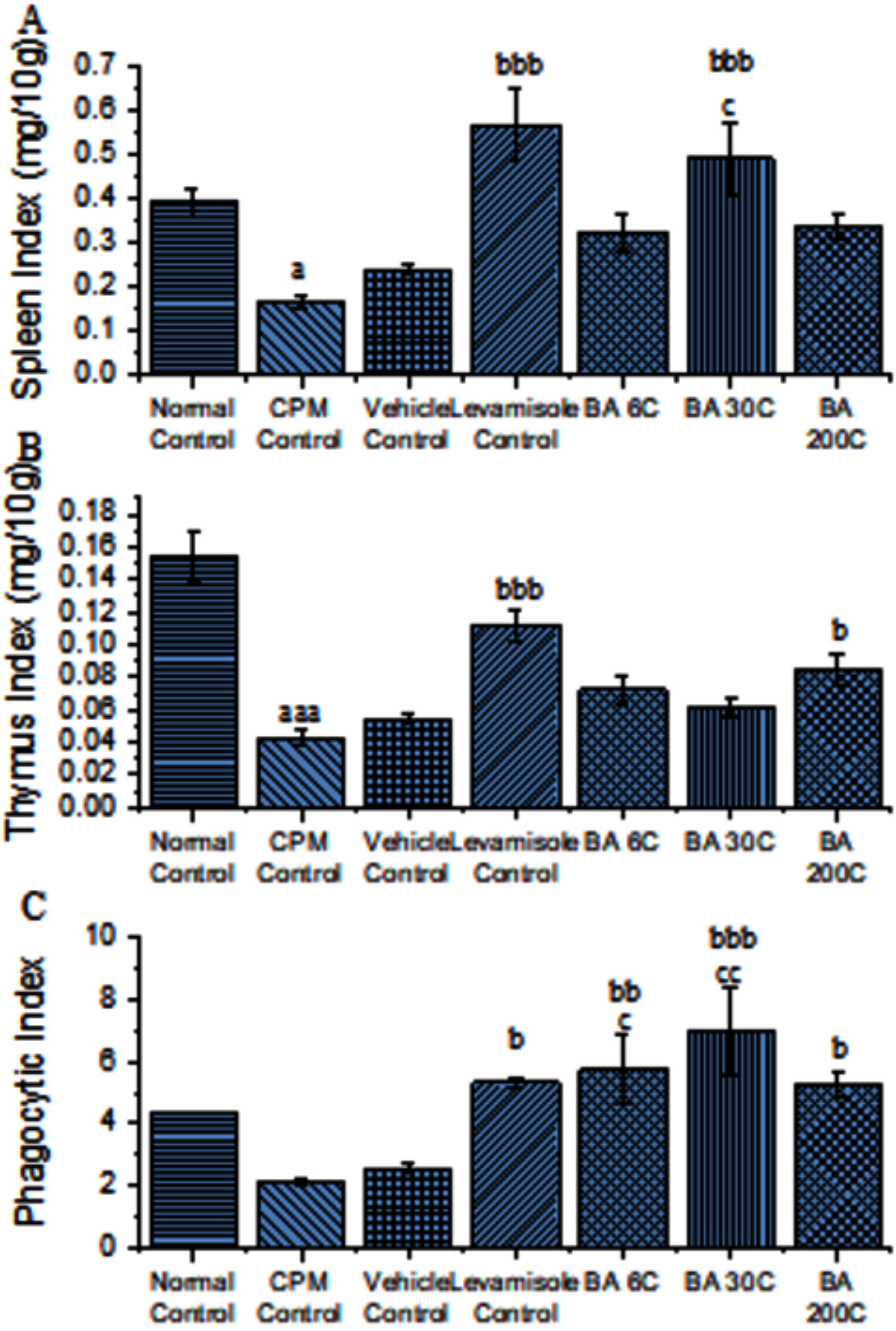

Fig 3 Click here to access/download;Figure;Fig 3.tif

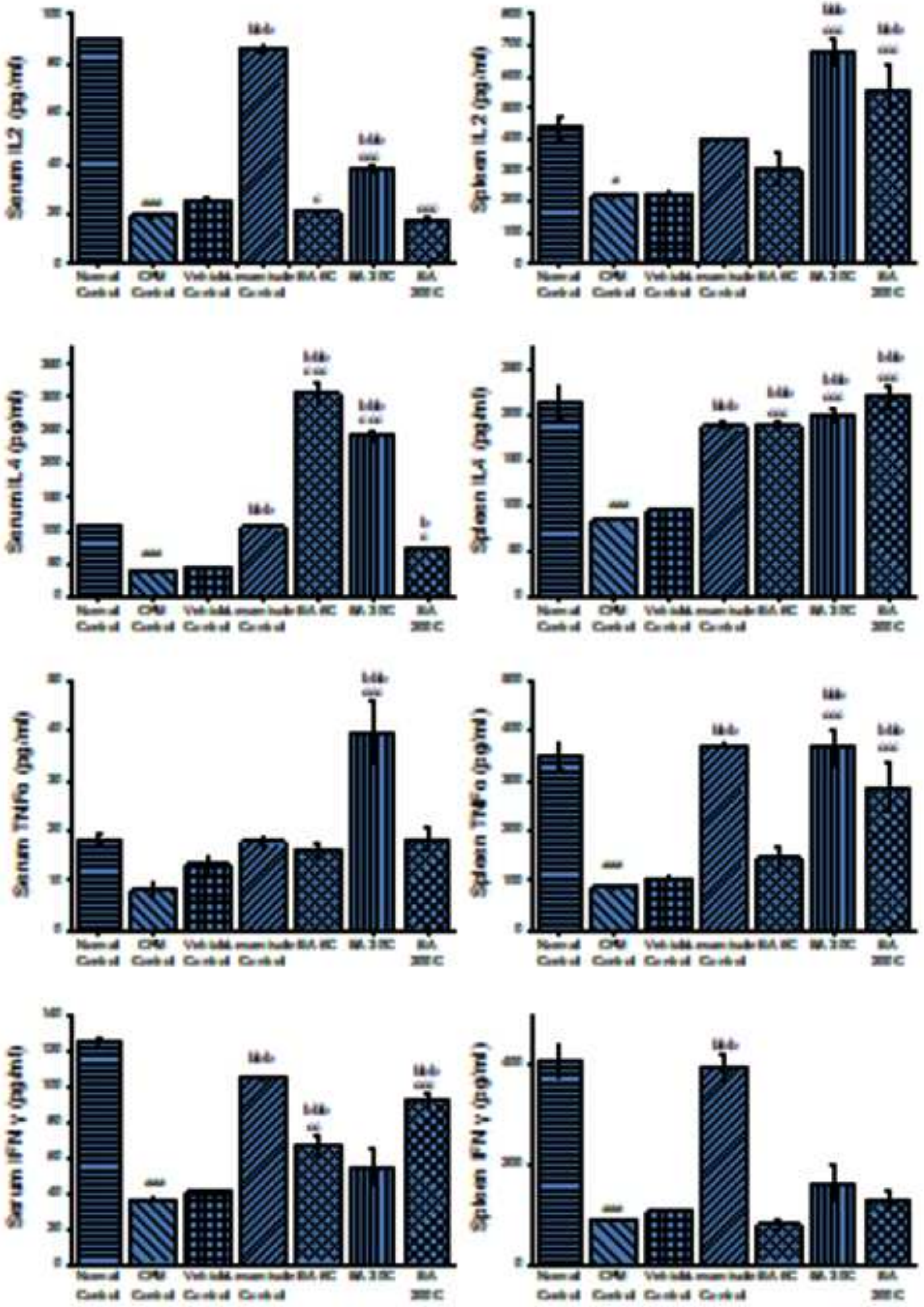

Fig 4

Click here to access/download;Figure;fIG 4.tif

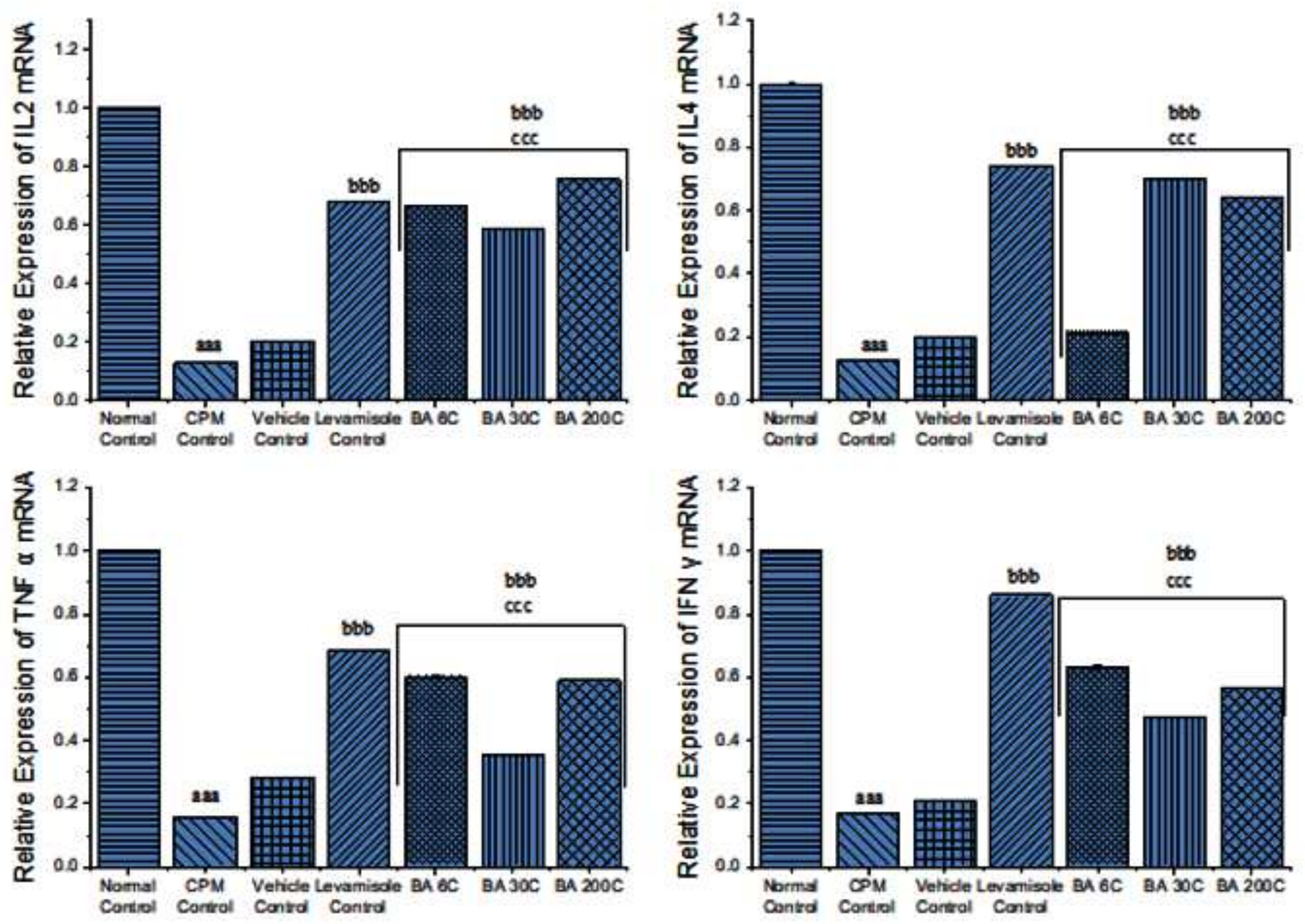

Fig 5

Click here to access/download;Figure;Fig 5.tif

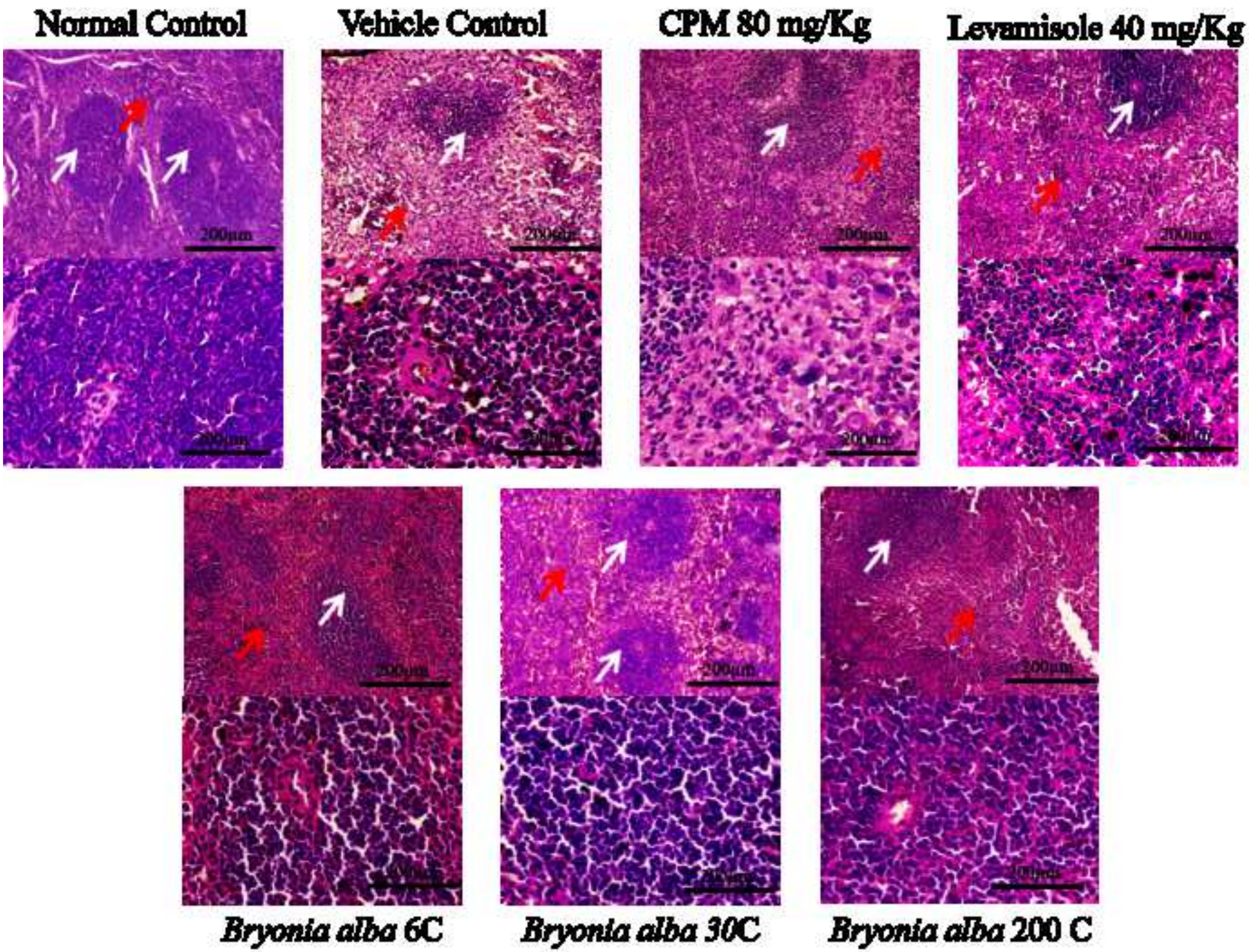

Supplement: Attachment — Submitted filename: PONE-D-24-16624_reviewer.pdf [file pone.0309756.s002.pdf]
