# Supplementary material for: Modulatory action of Bryonia alba on the immune system in cyclophosphamide induced immunosuppression in BALB/c mice
Source: PLoS One. 2024 Dec 31;19(12):e0309756. doi: 10.1371/journal.pone.0309756 (PMC11687816; doi:10.1371/journal.pone.0309756)
Supplement: Attachment — Submitted filename: Response to Reviewers.docx [file pone.0309756.s003.docx]

31-07-2024,

Kolkata.

To

The Editor-in-Chief,

PLOS ONE.

Dear Sir/Madam,

Re: Resubmission of Manuscript ID - **PONE-D-24-16624** entitled “**Modulatory Action of Bryonia alba on the Immune System in Cyclophosphamide Induced Immunosuppression in BALB/c Mice**”.

We are thankful for providing an opportunity to revise the Manuscript ID - PONE-D-24-16624 entitled " Modulatory Action of Bryonia alba on the Immune System in Cyclophosphamide Induced Immunosuppression in BALB/c Mice".

We value the time and energy that the editors and reviewers have invested in providing insightful commentary. Please find the amended manuscript, which incorporates the editor's and reviewer’s suggestions, together with thorough point-by-point responses to their comments. In this revision, we have revised the text to minimize technical and language errors. In order to address the suggestions made by editors and reviewers we have made inclusions and changes in the references 3-6, 9-21, 23, 24, and 26. However, if it still needs to meet the criteria required by the journal, we would be grateful if you kindly advise in this regard.

We wish this version will satisfy the editors and reviewers and meet all the standards of the journal. We welcome further constructive comments for the betterment of the manuscript if any.

Thank you,

Your Sincerely

Dr. G. V. Narasimha Kumar,

R.O. (Pharmacology), Scientist -1,

Dr. Anjali Chatterji Regional Research Institute for Homoeopathy,

Central Council for Research in Homoeopathy, Govt. of India,

Kolkata – 700035.

Contact:

Mobile: +91 9949102404

Email: narasimha.spsp@gmail.com

**Responses to Editor Comments:**

1. Please ensure that your manuscript meets PLOS ONE's style requirements, including those for file naming. The PLOS ONE style templates can be found at

https://journals.plos.org/plosone/s/file?id=wjVg/PLOSOne_formatting_sample_main_body.pdf and

https://journals.plos.org/plosone/s/file?id=ba62/PLOSOne_formatting_sample_title_authors_affiliations.pdf

**Response:** We have reviewed and ensured that our manuscript now fully complies with PLOS ONE's style requirements. The manuscript has been formatted according to the guidelines provided in the PLOS ONE style templates for both the main body and the title, authors, and affiliations sections. Additionally, all files have been renamed according to the specified naming conventions.

2. To comply with PLOS ONE submissions requirements, in your Methods section, please provide additional information regarding the experiments involving animals and ensure you have included details on (1) methods of sacrifice, and (2) efforts to alleviate suffering.

**Response:** We have updated the Methods section of our manuscript to include additional information regarding the experiments involving animals. Specifically, we have detailed the methods of sacrifice used in our study. Cervical dislocation was employed as the method of sacrifice, and this information has been included in the respective Methods section. Additionally, we have outlined the efforts taken to alleviate suffering, ensuring that all procedures were conducted in accordance with ethical guidelines to minimize discomfort and stress to the animals.

3. We note that the grant information you provided in the ‘Funding Information’ and ‘Financial Disclosure’ sections do not match. When you resubmit, please ensure that you provide the correct grant numbers for the awards you received for your study in the ‘Funding Information’ section.

**Response:** Thank you for bringing this to our attention. We have now updated both the ‘Funding Information’ and ‘Financial Disclosure’ sections with the correct and consistent grant numbers for the awards received for our study. This ensures that the information provided in both sections is accurate and aligned.

4. Thank you for stating the following financial disclosure:

[Central Council for Research in Homoeopathy, India sponsored the study under the Intramural Research Scheme (4-13/2019-20/CCRH/Tech/DS/Kolkata/DSU/3255 dated 18-03-2021).].

Please state what role the funders took in the study. If the funders had no role, please state: ""The funders had no role in study design, data collection and analysis, decision to publish, or preparation of the manuscript.""

If this statement is not correct you must amend it as needed.

Please include this amended Role of Funder statement in your cover letter; we will change the online submission form on your behalf.

**Response:** Thank you for your guidance. We have updated the financial disclosure statement to clearly state the role of the funders. The updated statement is: "The funders had no role in study design, data collection and analysis, decision to publish, or preparation of the manuscript." This amendment has been included in the financial disclosure section of the manuscript as well as in the cover letter.

5. We note that your Data Availability Statement is currently as follows: [All relevant data are within the manuscript and its Supporting Information files.]

Please confirm at this time whether or not your submission contains all raw data required to replicate the results of your study. Authors must share the “minimal data set” for their submission. PLOS defines the minimal data set to consist of the data required to replicate all study findings reported in the article, as well as related metadata and methods (https://journals.plos.org/plosone/s/data-availability#loc-minimal-data-set-definition).

For example, authors should submit the following data:

a. The values behind the means, standard deviations and other measures reported;
b. The values used to build graphs;
c. The points extracted from images for analysis.

Authors do not need to submit their entire data set if only a portion of the data was used in the reported study.

If your submission does not contain these data, please either upload them as Supporting Information files or deposit them to a stable, public repository and provide us with the relevant URLs, DOIs, or accession numbers. For a list of recommended repositories, please see https://journals.plos.org/plosone/s/recommended-repositories.

If there are ethical or legal restrictions on sharing a de-identified data set, please explain them in detail (e.g., data contain potentially sensitive information, data are owned by a third-party organization, etc.) and who has imposed them (e.g., an ethics committee). Please also provide contact information for a data access committee, ethics committee, or other institutional body to which data requests may be sent. If data are owned by a third party, please indicate how others may request data access.

**Response:** We have reviewed our Data Availability Statement and confirm that our submission contains all data required to replicate the results of our study. The following data have been included as Supporting Information files:

a. The values used to build graphs presented in the manuscript.

We have ensured that the minimal data set, as defined by PLOS ONE, is available to support replication of our study findings. If any additional data or information is required, please let us know, and we will be happy to provide it.

6. We note that Figure 5 in your submission contain copyrighted images. All PLOS content is published under the Creative Commons Attribution License (CC BY 4.0), which means that the manuscript, images, and Supporting Information files will be freely available online, and any third party is permitted to access, download, copy, distribute, and use these materials in any way, even commercially, with proper attribution. For more information, see our copyright guidelines: <http://journals.plos.org/plosone/s/licenses-and-copyright>.

We require you to either (1) present written permission from the copyright holder to publish these figures specifically under the CC BY 4.0 license, or (2) remove the figures from your submission:

a. You may seek permission from the original copyright holder of Figure 5 to publish the content specifically under the CC BY 4.0 license.

We recommend that you contact the original copyright holder with the Content Permission Form (http://journals.plos.org/plosone/s/file?id=7c09/content-permission-form.pdf) and the following text:

“I request permission for the open-access journal PLOS ONE to publish XXX under the Creative Commons Attribution License (CCAL) CC BY 4.0 (http://creativecommons.org/licenses/by/4.0/). Please be aware that this license allows unrestricted use and distribution, even commercially, by third parties. Please reply and provide explicit written permission to publish XXX under a CC BY license and complete the attached form.”

Please upload the completed Content Permission Form or other proof of granted permissions as an ""Other"" file with your submission.

In the figure caption of the copyrighted figure, please include the following text: “Reprinted from [ref] under a CC BY license, with permission from [name of publisher], original copyright [original copyright year].”

b. If you are unable to obtain permission from the original copyright holder to publish these figures under the CC BY 4.0 license or if the copyright holder’s requirements are incompatible with the CC BY 4.0 license, please either i) remove the figure or ii) supply a replacement figure that complies with the CC BY 4.0 license. Please check copyright information on all replacement figures and update the figure caption with source information. If applicable, please specify in the figure caption text when a figure is similar but not identical to the original image and is therefore for illustrative purposes only.

**Response:** We understand the concern regarding Figure 5. Figure 5 contains a total of 14 different images, which were taken using a BX43 Olympus microscope with a DP22 camera at 1920*1440 pixels through 10x and 40x objective lenses and were merged accordingly. We declare that the images used are original and belong to the Department of Pharmacology, Dr. Anjali Chatterjee Regional Research Institute for Homoeopathy, Kolkata. Figure 5 metadata file has been attached for your reference and perusal.

7. Please include a caption for figure 5.

**Response:** We have included the caption for figure 5.

8. Please review your reference list to ensure that it is complete and correct. If you have cited papers that have been retracted, please include the rationale for doing so in the manuscript text, or remove these references and replace them with relevant current references. Any changes to the reference list should be mentioned in the rebuttal letter that accompanies your revised manuscript. If you need to cite a retracted article, indicate the article’s retracted status in the References list and also include a citation and full reference for the retraction notice.

**Response:** We have thoroughly reviewed our reference list to ensure its completeness and accuracy. We confirm that no retracted articles have been cited in our manuscript. This information has been included in the rebuttal letter accompanying our revised manuscript. If any updates or corrections were needed, they have been made accordingly.

**Responses to Reviewer 1 Comments:**

**Comment: 1**

I suggest that the author pays attention to the bibliographical references in the introduction. I consider the references used to be somewhat outdated for the publication, I try citations that have been published for more than 14 years.

**Response:** Thank you for your valuable feedback. We acknowledge that some of the references in the introduction are outdated. We have updated the introduction with more recent and relevant studies to support our claims. This includes incorporating studies from the past decade to provide a current perspective on the topic.

**Responses to Reviewer 2 Comments:**

**Comment: 1**

In the introduction, some improvements can be implemented to strengthen the scientific basis and clarity of the text. Initially, it would be beneficial to update the references used to support claims about homeopathy and Bryonia alba, incorporating more recent and robust studies to increase the credibility of the information presented. Furthermore, it is important to detail the evidence mentioned in the introduction. Although several studies on the use of BA are cited, there is a lack of specifications on study design, sample sizes, and specific results. Including more details could strengthen the claims and provide a clearer view of the current scientific landscape.

**Response:** Thank you for your insightful comments. We have updated the references in the introduction to include more recent and robust studies, which enhances the credibility of our information on homeopathy and Bryonia alba. Additionally, we have provided more detailed descriptions of the cited studies, including their design, sample sizes, and specific results, to enhance the scientific basis and clarity of the text.

**Comment: 2**

To improve the accessibility of the text, it could be useful to briefly explain some technical terms,

such as "immunosuppression" and "homeopathic potencies", making it more understandable for non-specialist readers. In addition, small grammatical and concordance errors must be reviewed to ensure the fluidity and accuracy of the text.

**Response:** We have added brief explanations for technical terms like "immunosuppression" and "homeopathic potencies" to enhance the accessibility of the text for non-specialist readers. Additionally, we have reviewed and corrected small grammatical and concordance errors to ensure better fluidity and accuracy.

**Comment: 3**

Regarding the mechanisms of action of BA, the introduction mentions its immunomodulatory and antioxidant characteristics, but does not offer a detailed explanation of the possible underlying biological mechanisms. A deeper analysis of these mechanisms would not only increase understanding but also reinforce the credibility of the conclusions presented. The rationale for the study can also be expanded. While the introduction states that the immunostimulating properties of BA in situations of immunosuppression have not been adequately explored, it would be useful to explain in more detail why this gap in knowledge is relevant and what the potential clinical impacts of this research are. Finally, discussing the challenges inherent in researching homeopathic treatments, such as the variability of results and the difficulty of replication, would provide a more balanced and critical view of the study, offering a more complete perspective for readers.

**Response:** We agree with your suggestion. In the revised manuscript, we have provided a more detailed explanation of the possible underlying biological mechanisms of BA's immunomodulatory and antioxidant characteristics. Additionally, we have expanded the rationale for the study, explaining the relevance of the knowledge gap and the potential clinical impacts. We have also discussed the challenges inherent in researching homeopathic treatments, such as variability of results and difficulty of replication, to provide a more balanced and critical view of the study.

**Comment: 4**

In the methodology, some improvements can be suggested to strengthen the robustness of the study. First, it would be beneficial to supplement the references used (e.g., Singh et al., 2019; Jahnavi et al., 2019) with more recent studies or updated literature reviews that solidify the scientific basis for the doses and procedures employed. Additionally, the list of reagents and chemicals could be expanded to include all critical materials used in the study, such as ELISA kits, reagents for RNA and cDNA, and the specific equipment used in PCR and microscopy analyses. The justification for the choice of dose, although it mentioned the conversion of the dose from albino rats to albino mice, could be more robust. It would be helpful to provide better reasoning, perhaps with additional references or a more detailed explanation of the conversion process used to determine the final volume (40 μL/100 g). Furthermore, some minor procedural details, such as the exact protocol for retro-orbital blood collection, could be more thoroughly described. This would ensure that all procedures can be replicated accurately, promoting the consistency and reliability of the results obtained.

**Response:** Thank you for your valuable feedback. In the revised manuscript, we have updated the references in the methodology section with more recent studies and literature reviews. We have also expanded the list of reagents and chemicals to include all critical materials used in the study. The justification for the choice of dose has been strengthened with additional references and a more detailed explanation of the conversion process. Furthermore, we have provided a more thorough description of minor procedural details, such as the exact protocol for retro-orbital blood collection, to ensure accurate replication of procedures.

**Comment: 5**

The discussion recognizes the limitation of using an animal model to simulate chemotherapy-induced immunosuppression, which may not fully replicate human immune responses. This recognition is important as it highlights the need for clinical studies in humans to validate these findings. Research could benefit from further exploration of the molecular mechanisms by which BA exerts its immunomodulatory effects. Investigating cellular signaling pathways and molecular interactions would provide a more complete understanding of BA's modes of action. The discussion addresses the use of alcohol as a vehicle for BA and its lack of impact on the observed immunomodulatory properties, which is relevant to ensure that the beneficial effects are due to the BA and not the vehicle. Comparison with Levamisole, a known immunomodulator, strengthens the results, demonstrating that BA has comparable efficacy, which is encouraging for the validation of BA as a potential therapeutic agent.

**Response:** Thank you for your constructive feedback. As discussed in the limitations, we are positively looking forward to extend the work to find out the molecular signaling pathways of BA.

**Comment: 6**

The conclusion effectively summarizes the main results, such as increased levels of RBC, WBC, spleen indices and cytokines (IL-2, IL-4, TNF-α, IFN-γ) in serum and spleen, reinforcing the evidence that BA has a relevant immunomodulatory action. While recognizing that the mechanism of action of BA is still unknown, it would be useful to suggest specific methods or approaches to explore these mechanisms in future research, adding value and direction to studies. It is important to reinforce caution when generalizing results from animal models to humans, contextualizing the findings within the limits of preclinical research. The conclusion could include a brief reference to previous studies on immunomodulation, better situating the findings within the broader body of scientific literature. Furthermore, explicitly suggesting clinical trials to validate the effects in humans would be valuable, showing a clear path to practical application of the results.

**Response:** Thank you for your constructive feedback. We had modified the conclusion in the manuscript.
